# Derivation of a Precise and Consistent Timeline for Antibiotic Development

**DOI:** 10.3390/antibiotics11091237

**Published:** 2022-09-12

**Authors:** Henry L. Stennett, Catherine R. Back, Paul R. Race

**Affiliations:** 1 School of Biochemistry, University of Bristol, University Walk, Bristol BS8 1TD, UK; 2 BrisSynBio Synthetic Biology Research Centre, Tyndall Avenue, Bristol BS8 1TQ, UK

**Keywords:** antibiotic, timeline, discovery, clinical, resistance, development

## Abstract

Antibiotic resistance is a global health crisis. New classes of antibiotics that can treat drug-resistant infections are urgently needed. To communicate this message, researchers have used antibiotic development timelines, but these are often contradictory or imprecise. We conducted a systematic literature review to produce an antibiotic timeline that incorporates the dates of discovery, first use, and initial reports of the emergence of resistance for the 38 classes of clinically used antibiotics. From our timeline, we derive lessons for identifying new antibiotics that are less prone to resistance. These include a required focus on molecules that exhibit multiple modes of action, possess unusually long ‘resistance windows’, or those that engage cellular targets whose molecular architectures are at least in part decoupled from evolutionary pressures. Our analysis also further highlights the importance of safeguarding antibiotics as a mechanism for mitigating the development of resistance. We have made our data and sources freely available so that the research community can adapt them to their own needs.

## 1. Introduction

Antibiotic resistance—bacterial infections that no longer respond to the drugs used to treat them—is a global health crisis of growing concern [1,2]. The demand for existing antibiotics is too high, which drives the evolution of resistance in pathogens [2,3,4]. The supply of new classes of antibiotics that can treat drug-resistant infections is too low, which leaves us with few treatment options for the most serious infections [5,6,7]. To communicate the scale of the problem, researchers have used graphical timelines that show how the rate of antibiotic discovery has slowed in recent times. These timelines are valuable tools for science communication, but often disagree with one another and lack clarity.

In previous reviews, the definitions used for the date of the discovery of an antibiotic, its first clinical use, and the emergence of resistance to it, either are not disclosed or lack internal consistency. Examples include the non-disclosure of methods used to generate a timeline for antibiotic discovery [5]; inconsistency in the definition of when an antibiotic was introduced into the clinic [8]; not defining the “year of discovery” or “year of introduction” categories and employing a scale with a resolution of decades [9]; and the CDC’s 2013 Antibiotic Threats Report, which includes an antibiotic resistance timeline “based on early reports in the literature”, but with a lack of clarity as to whether this refers to in vitro, in vivo, or clinical data [10].

**Figure 1:**
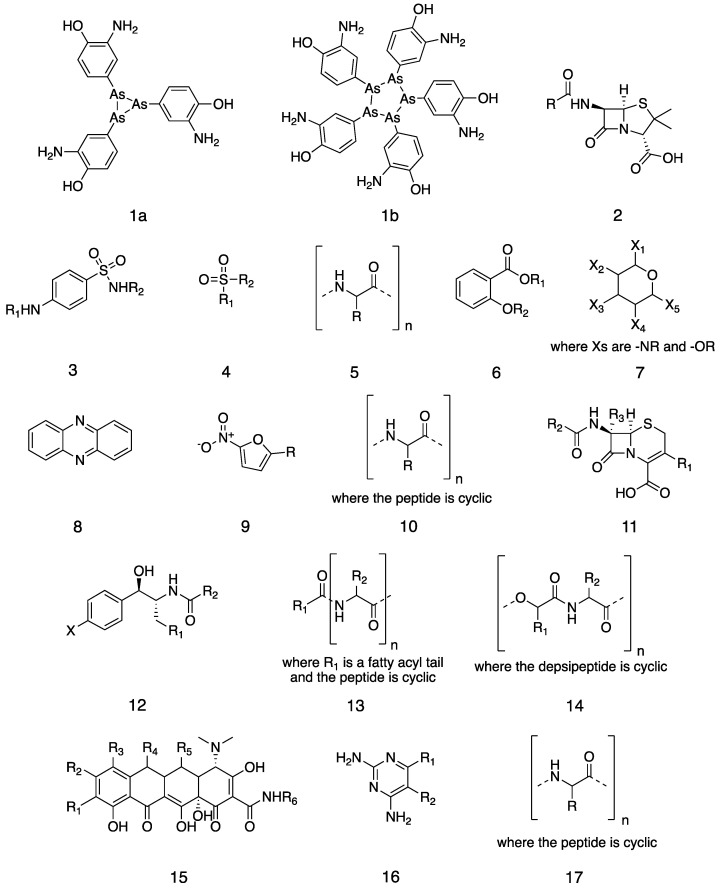

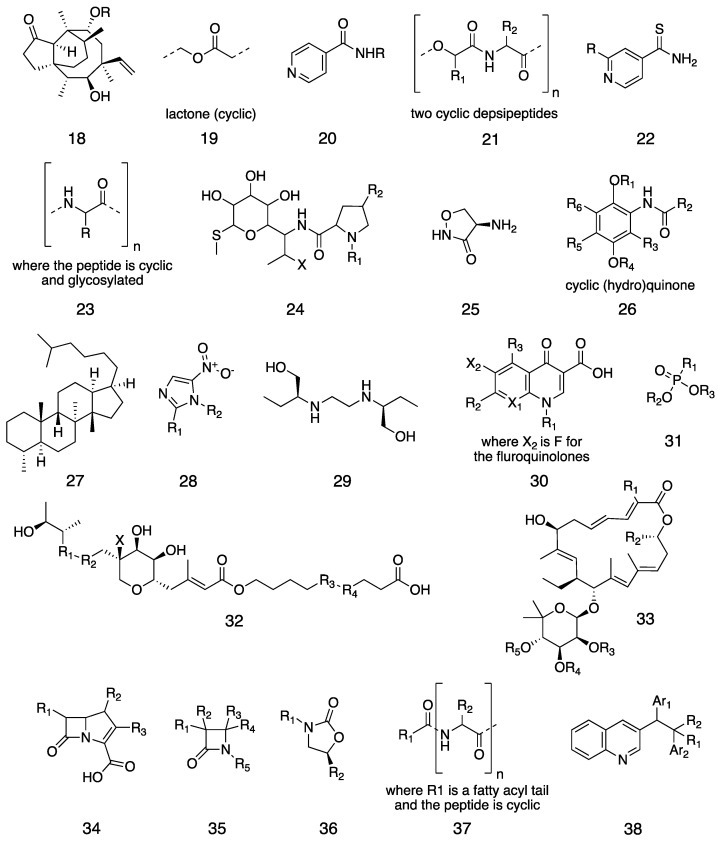
Chemical structures of the 38 classes of antibiotics. (**1**) Arsphenamine in its (**1a**) trivalent and (**1b**) pentavalent forms. General chemical structures of (**2**) a penicillin, (**3**) a sulfonamide, (**4**) a sulphone, (**5**) a polypeptide, (**6**) a salicylate, (**7**) an aminoglycoside, (**8**) a phenazine, (**9**) a nitrofuran, (**10**) a cyclic peptide, (**11**) a cephalosporin, (**12**) an amphenicol, (**13**) a polymyxin, (**14**) an enniatin, (**15**) a tetracycline, (**16**) a diaminopyrimidine, (**17**) a tuberactinomycin, (**18**) a pleuromutilin, (**19**) a macrolide, (**20**) a nicotinamide, (**21**) a streptogramin, (**22**) a thioisonicotinamide, (**23**) a glycopeptide, (**24**) a lincosamide, (**25**) a cycloserine, (**26**) an ansamycin, (**27**) a fusidane, (**28**) a nitroimidazole, (**29**) ethambutol, (**30**) a quinolone, (**31**) a phosphonate, (**32**) a mupirocin, (**33**) a lipiarmycin, (**34**) a carbapenem, (**35**) a monobactam, (**36**) an oxazolidinone, (**37**) a lipopeptide, and (**38**) a diarylquinoline.

We conducted a systematic literature review to produce a more precise timeline for antibiotic discovery, introduction, and resistance. Antibiotics can be classified by their origins, structures, and mechanisms of action. We took the 38 classes of antibiotics in clinical use (Figure 1), as defined by Hutchings et al. [8], and consistently applied the following definitions to them:

1. **Discovery:** when a compound or extract—not merely an organism—was first reported to have antibiotic activity. In some cases, this was decades after the compound was first discovered or synthesized.
2. **First clinical use:** the first use of the antibiotic to treat a bacterial infection: in modern terms, a phase II trial. Clinical studies of tolerance and toxicity are not included. The approval of an antibiotic for human use is not used because several of these drugs predate regulatory bodies.
3. **Resistance:** the first report of clinically isolated bacteria resistant to the antibiotic.

We hope that our timeline will be a useful resource to the antibiotic research and public health communities. We have included our data and sources so that the timeline can be verified, updated, and modified.

## 2. Results

**Figure 2:**
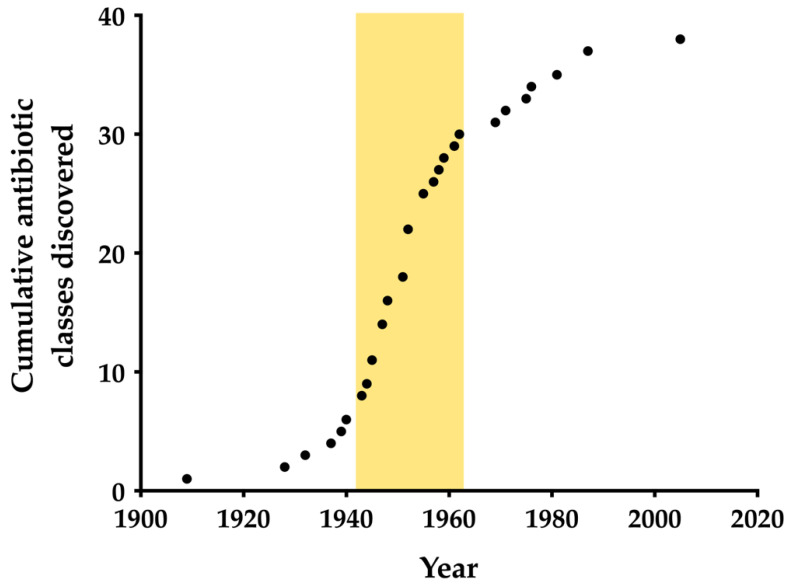
The cumulative discovery of the 38 classes of clinically used antibiotics. The Golden Age of discovery is highlighted in yellow.

Figure 2 emphasizes the stark reduction in the antibiotic discovery rate after the “Golden Age”, the most prolific period of antibiotic research [11,12]. In fact, the rate of discovery is now at its lowest since the first antibiotic, arsphenamine, was discovered in 1909. The Golden Age is usually roughly defined as 1940–1960, beginning with the discovery of streptomycin [8]. Extending the linear part of the sigmoidal discovery curve in Figure 1 allows us to better define the Golden Age as 1943–1962, when streptomycin and the quinolones were discovered, respectively. A 2011 review in *Clinical Microbiology Reviews* defined the “discovery void”, during which no new antibiotic classes have been discovered, as starting from 1987, and several sources have repeated this claim [5,13,14,15]. However, the diarylquinolines were FDA-approved the year after this review, and thus this definition requires revision [16].

**Figure 3:**
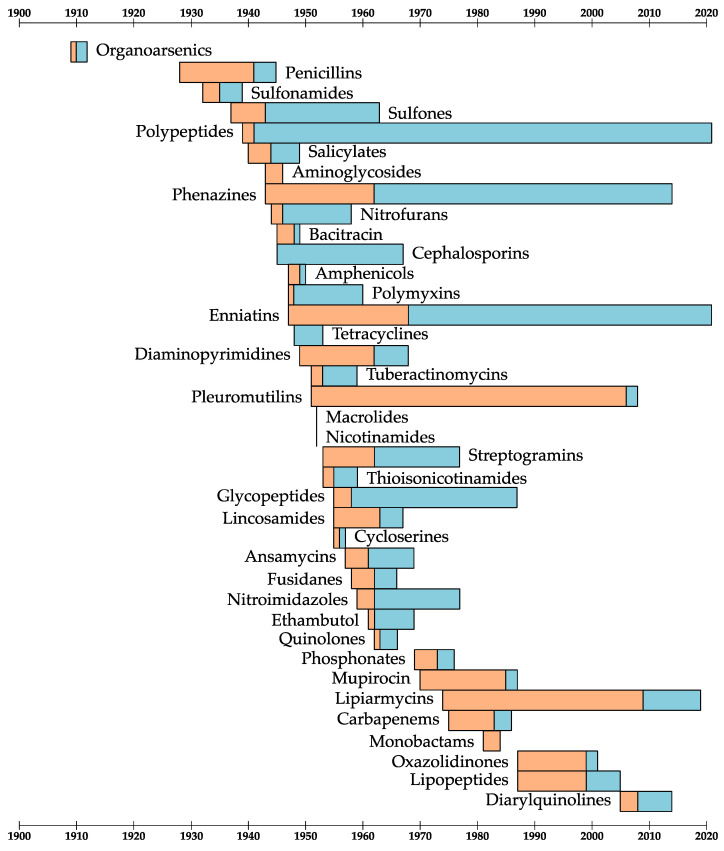
A timeline for the discovery, first clinical use of, and first report of clinical resistance to the 38 classes of antibiotics. For each antibiotic class, the orange bars are the “development windows” and the blue bars are the “resistance windows”.

Figure 3 shows the discovery, first clinical use, and resistance dates for the 38 antibiotic classes. From these dates, we can define two periods of time:

1. **The development window:** how long after its discovery the antibiotic was first used in the clinic.
2. **The resistance window:** how long after its first use clinical resistance was reported.

There are some obvious outliers in this analysis. The antibiotics with long development windows were either technically challenging to optimize or shelved because they were not considered to be promising drugs until the antibiotic resistance crisis worsened [17,18,19,20]. Five new antibiotic classes have been approved for human use by the FDA in this century: oxazolidinones (2000), lipopeptides (2003), pleuromutilins (2007), diarylquinolones (2007), and lipiarmycins (2011). Three of these were abandoned early in their development because of adverse side effects [16,17,21]. The diarylquinolones carry a black box warning—the strongest warning that the FDA requires—because of their significant life-threatening side effects [16]. The lipiarmycins and pleuromutilins were first approved for human use long after their discovery: 36 and 56 years, respectively [17,22].

More promisingly, there are a few examples of antibiotics with unusually long resistance windows, from which we can derive some lessons for designing or identifying “resistance-proof” antibiotics [23,24].

### 2.1. Multiple Targets

The polypeptide antibiotic tyrothricin has only been used topically, which is likely part of the reason for its long resistance window [25]. However, even after decades of use, no clinical resistance to the antibiotic has been seen and significant resistance cannot be induced in vitro [25,26]. Wenzel et al. interrogated the antibiotic mechanism of tyrothricin and found that even though its component peptides are highly similar in sequence, they have different mechanisms of action [27]. Their combined effects are to damage DNA, increase membrane permeability, decrease membrane fluidity, and delocalize membrane proteins [27]. This attack on multiple fronts is difficult for bacteria to defend against and makes tyrothricin a natural combination therapy [28]. Clinical phenazine resistance is also extremely rare, although it has been induced in vitro [29,30]. Like tyrothricin, these antibiotics likely have multiple mechanisms of action, which makes resistance more difficult to evolve [31,32]. Identifying new antibiotics with multiple mechanisms of action, or using multiple antibiotics as combination therapies, is likely to slow the development of resistance [28].

### 2.2. “Resistance-Proof” Targets

Glycopeptides such as vancomycin bind D-Ala-D-Ala residues at the ends of glycan chains, preventing the binding of peptidoglycan biosynthetic enzymes [33]. This mode of action targets a structural component of the cell that is not directly genetically encoded, so it is difficult to evolve resistance by mutating the target [34]. Furthermore, glycopeptides do not have to enter the cell to act, which means that resistance cannot evolve by reduced permeability to or modification of the antibiotics [34]. Resistance to glycopeptides did not arise in pathogens directly: the self-resistance genes were transferred from the producing microorganisms to pathogens [34]. New antibiotics with similar targets are likely to be “resistance-proof” [23].

### 2.3. Low Use

The long resistance window for sulfones is probably due to their main indication as drugs for leprosy [35]. Leprosy is a neglected tropical disease and there are many gaps in our understanding of it [36]. Furthermore, sulfones are only weakly antibacterial against *Mycobacterium leprae*, which made resistance to these antibiotics more difficult to definitively prove [37]. Enniatins are thought to act as ionophores, collapsing ion gradients across membranes in general [38]. In vivo resistance to ionophores has been recorded and can occur by enzymatic degradation or exclusion of the compounds from the cell [39]. Presumably, such mechanisms are also possible for the enniatins, and their limited use due to their cytotoxicity has delayed the onset of resistance [40,41]. The most important lesson for safeguarding antibiotics is that reducing their use will slow the development of resistance [4].

## 3. Discussion

This work represents the first comprehensive and consistent timeline for antibiotic discovery, development, and resistance. It should prove useful for communicating the alarmingly low number of new antibiotic classes that are reaching the clinic, and we have also shown how the data can be used to identify antibiotic classes for which resistance is more difficult to evolve. Our findings reveal a correlation between pharmacophore novelty and a reduced ‘development window’, and also serve to highlight the importance of prioritizing molecules with expanded ‘resistance windows’ to ensure the long-term safeguarding of antibiotics. By making our data fully available, and our methods transparent, we hope that future researchers can use and adapt our timeline for their own science communication.

## 4. Materials and Methods

We conducted a systematic literature review by searching the Web of Science Core Collection and PubMed databases for the names of antibiotic classes or their first members, and found the earliest dates of discovery, use, and resistance, defined in Section 1. Table 1 shows the data used to make these timelines, with sources for each data point.

## Figures and Tables

**Table 1:** The data used to generate Figure 1 and Figure 2.

Antibiotic Class	Discovery Date	Clinical Use Date	Resistance Date
Organoarsenics	1909 [42]	1910 [42]	1912 [43]
Penicillins	1928 [44]	1941 [45]	1945 [46]
Sulfonamides	1932 [42]	1935 [47]	1939 [48]
Sulfones	1937 [49]	1943 [50]	1963 [35]
Polypeptides	1939 [51]	1941 [52]	N/A ^1^ [25]
Salicylates	1940 [53]	1944 [54]	1949 [55]
Aminoglycosides	1943 [56]	1946 [57]	1946 [58]
Phenazines	1943 [59]	1962 [60]	2014 [61]
Nitrofurans	1944 [62]	1946 [63]	1958 [64]
Bacitracin	1945 [65]	1948 [66]	1949 [67]
Cephalosporins	1945 [68]	1945 [68]	1967 [69]
Amphenicols	1947 [70]	1949 [71]	1950 [72]
Polymyxins	1947 [73]	1948 [74]	1960 [75]
Enniatins	1947 [76]	1968 [77]	N/A ^1^ [78]
Tetracyclines	1948 [79]	1948 [80]	1953 [81]
Diaminopyrimidines	1948 [82]	1962 [83]	1968 [84]
Tuberactinomycins	1951 [85]	1953 [86]	1959 [87]
Pleuromutilins	1951 [88]	2006 [89]	2008 [90]
Macrolides	1952 [91]	1952 [92]	1952 [93]
Nicotinamides	1952 [94,95]	1952 [96]	1952 [97]
Streptogramins	1952 [98]	1962 [99]	1977 [100]
Thioisonicotinamides	1952 [101]	1955 [102]	1959 [101]
Glycopeptides	1955 [103]	1958 [104]	1987 [105]
Lincosamides	1955 [106]	1963 [107]	1967 [108]
Cycloserines	1955 [109]	1956 [110]	1957 [111]
Ansamycins	1957 [112]	1961 [113]	1969 [114]
Fusidanes	1958 [115,116,117]	1962 [118]	1966 [119]
Nitroimidazoles	1959 [120]	1962 [121]	1978 [122]
Ethambutol	1961 [123]	1962 [124]	1969 [125]
Quinolones	1962 [126]	1963 [127]	1966 [128]
Phosphonates	1969 [129]	1974 [130]	1977 [131]
Mupirocin	1971 [132]	1985 [133]	1987 [134]
Lipiarmycins	1975 [135]	2009 [136]	2019 [137]
Carbapenems	1976 [138]	1983 [139]	1986 [140]
Monobactams	1981 [141]	1984 [142]	1984 [143]
Oxazolidinones	1987 [144]	1999 [145]	2001 [146]
Lipopeptides	1987 [147]	1999 [148]	2005 [149]
Diarylquinolines	2005 [150]	2008 [151]	2014 [61]

^1^ Not applicable—clinical resistance is yet to be identified.

## Data Availability

No new data were created or analyzed in this study. Data sharing is not applicable to this article.
